# Substrate texture affects female cricket walking response to male calling song

**DOI:** 10.1098/rsos.172334

**Published:** 2018-03-07

**Authors:** E. J. Sarmiento-Ponce, M. P. F. Sutcliffe, B. Hedwig

**Affiliations:** 1 Department of Zoology, University of Cambridge, Downing Street, Cambridge CB2 3EJ, UK; 2 Department of Engineering, University of Cambridge, Trumpington Street, Cambridge CB2 1PZ, UK

**Keywords:** ground-living insects, insect walking, tarsal claws, biomechanics, depth profile, contact force measurements

## Abstract

Field crickets are extensively used as a model organism to study female phonotactic walking behaviour, i.e. their attraction to the male calling song. Laboratory-based phonotaxis experiments generally rely on arena or trackball-based settings; however, no attention has been paid to the effect of substrate texture on the response. Here, we tested phonotaxis in female *Gryllus bimaculatus*, walking on trackballs machined from methyl-methacrylate foam with different cell sizes. Surface height variations of the trackballs, due to the cellular composition of the material, were measured with profilometry and characterized as smooth, medium or rough, with roughness amplitudes of 7.3, 16 and 180 µm. Female phonotaxis was best on a rough and medium trackball surface, a smooth surface resulted in a significant lower phonotactic response. Claws of the cricket foot were crucial for effective walking. Females insert their claws into the surface pores to allow mechanical interlocking with the substrate texture and a high degree of attachment, which cannot be established on smooth surfaces. These findings provide insight to the biomechanical basis of insect walking and may inform behavioural studies that the surface texture on which walking insects are tested is crucial for the resulting behavioural response.

## 1. Introduction

Arthropods are the most diverse group in the animal kingdom. One of the reasons is that species evolved abilities to move in different environments. Specialized structures on the tarsi of an insect leg allow efficient locomotion on a variety of substrates. Adhesive pads provide mechanical aids for walking on flat and slippery surfaces. Their function has been extensively studied in stick insects, cockroaches, bush crickets and ants [1–5]. However, little is known about the efficiency and performance of locomotion in ground-living insects, such as crickets, that are in constant contact with small particles such as sand and soil; they lack adhesive pads and present instead mechanical structures such as tarsal spurs, spines and claws [6].

The legs of field crickets are adapted to engage with the ground surface, when female crickets walk over different substrates in order to reach a singing male. For more than 30 years, behavioural studies of cricket phonotactic behaviour have been performed under laboratory conditions. Arena experiments used sand [7–9], paper sheets [10] or plastic sheets [11] as substrate. Trackball systems [12–16] have been used to analyse phonotactic responses and to infer the velocity and direction of the walking insect from the movement of the trackball. Polystyrene [17,18], hollow styrofoam [14] and Rohacell [19,20] are materials which have been used to build trackballs. Little or no detail is usually given regarding the surface structure of the arenas or trackballs used [13,21,22]; however, we do not yet understand how the surface texture could impact on insect walking. Here, we tested the phonotactic behaviour of female field crickets (*Gryllus bimaculatus*) on a trackball system; when responding to an attractive male calling song, a contact force experiment was also performed. We used three different trackball textures, in order to test and measure the impact that different surfaces have on their behavioural performance.

## 2. Material and methods

### 2.1. Animals and trackball system

Female crickets *G. bimaculatus* were taken from a colony at the University of Cambridge and kept on woodchips in 1.5 l plastic containers at 25–28°C with a 12 L : 12 D cycle. They were fed protein-rich diet and water. After the final moult, a metal pin (32 mg; cricket 1.2 g) was attached vertically to the third thoracic tergite. An open-loop trackball system was used for phonotaxis tests [19,20]; experiments were run in the dark at a temperature of 25–28°C. A total of 25 responsive females were used between 7 and 24 days after the final moult; they were tethered on the thorax and placed on a trackball in natural walking position.

### 2.2. Analysis of phonotactic walking behaviour and sound stimuli

**Figure 1.**
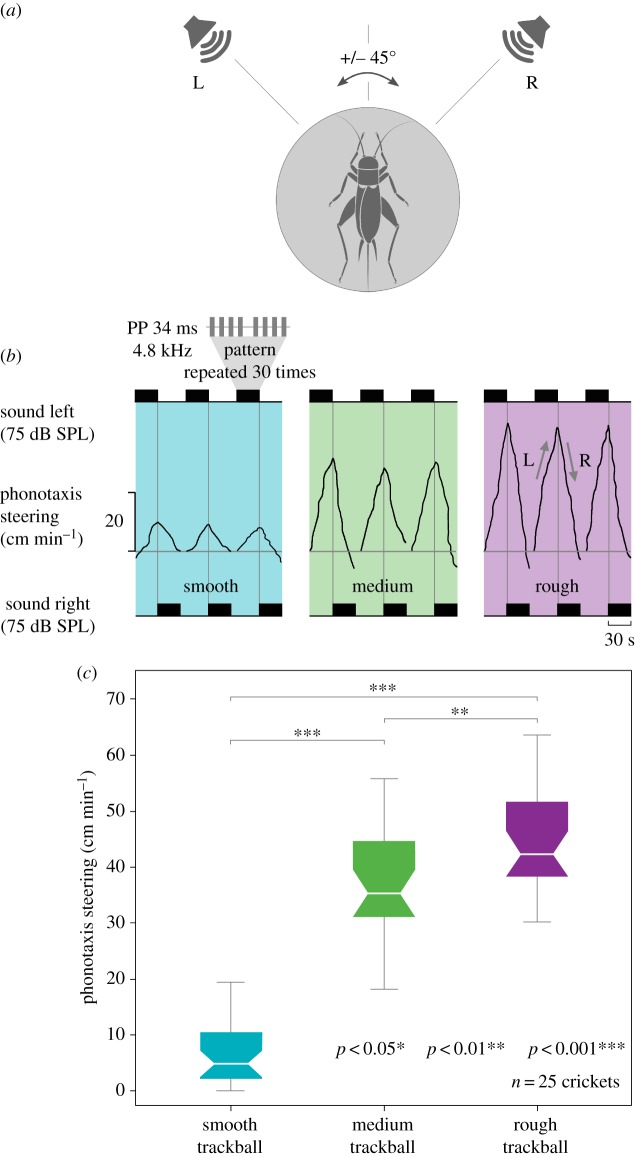
Female phonotactic steering on three different trackball surfaces. (*a*) Cricket on a trackball with speakers located to the left and right at 45°. (*b*) Exemplary phonotactic response for the smooth (blue), medium (green) and rough (purple) trackball surfaces. A male calling song (frequency of 4.8 kHz, 75** **dB SPL and 34** **ms pulse period) is repeatedly presented for 30** **s from the left and right side, as indicated by the black rectangles, including a 10 s gap to avoid a carry-on effect. Phonotaxis is demonstrated by steering of the female towards the left (L, upwards), and then to the right (R, downwards) as the active speaker changes. (*c*) Mean phonotactic response of *n* = 25 female crickets walking on different trackball surfaces, including Wilcoxon signed-rank analysis, indicating the behavioural responses on each trackball.

Computer-generated calling songs were presented by two speakers (Neo 13s, Sinus live, Conrad Electronics, Hirschau, Germany) in front of the cricket at a distance of 57 cm and at an angle of 45° (figure 1*a*) to the left and right of the animal's long axis. Sound intensities were 75 dB SPL relative to 10^−5^ Nm^−2^ at the position of the cricket [19,20]. The standard calling song sound pattern had a frequency of 4.8 kHz, 75 dB SPL and 34 ms pulse period, and in one test was presented three times for 30 s from the left and 30 s from the right side (figure 1*b*). The walking component towards the active speaker (i.e. the lateral deviation) was measured from the trackball rotations and the overall deviation towards the sound pattern was calculated for the duration of 1 min. Tests were performed inside a sound-proofed chamber; each cricket was tested three to five times on each of the different trackballs, and hence the lateral deviation was averaged. The lateral deviation is sufficient to measure phonotactic behaviour, the animals do not rotate while they are walking. In addition, the translation velocity does not add a crucial component to the characterization of phonotactic steering [19].

### 2.3. Measurements of force and claws

**Figure 2.**
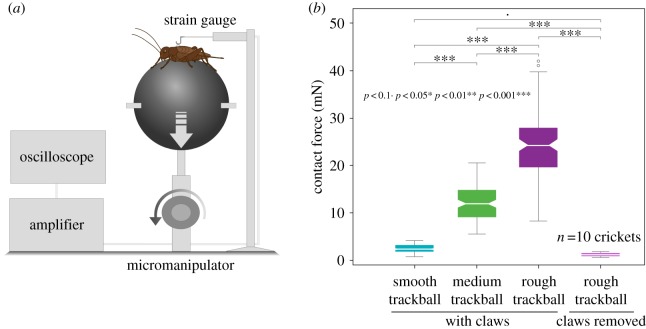
Contact force measurements of female crickets standing on different trackballs. (*a*) Crickets were attached to a strain gauge, while the trackball was lowered until the animals lost contact with the surface. (*b*) Contact forces between the cricket feet and the trackball on the smooth, medium and rough trackball surfaces and Wilcoxon signed-rank test of the contact forces, with crickets with intact claws and crickets without claws.

Contact force measurements between the trackball surface and the cricket feet were performed on 10 females with intact claws, and 10 females in which the claws were removed. Claws were carefully removed by cutting them with precision scissors, the tarsi of the females were not damaged in the process. A holder was attached with bees wax to the thorax at one end and to a calibrated strain gauge at the other. Crickets were positioned on the different trackballs and a micromanipulator lowered the trackball until the contact with the tarsi was lost (figure 2*a*; electronic supplementary material, Video S2). The micromanipulator was manually lowered 2.3 cm in less than 1 s. The strain gauge recorded the force required to overcome the contact between the tarsi and the trackball. It was calibrated with a series of weights (1–14 g; electronic supplementary material, figure S1), providing a linear regression (*y *= 45.72*x* + 14.97; *R*^2 ^= 0.996). Ten trials were measured for each cricket and each trackball surface.

**Figure 3.**
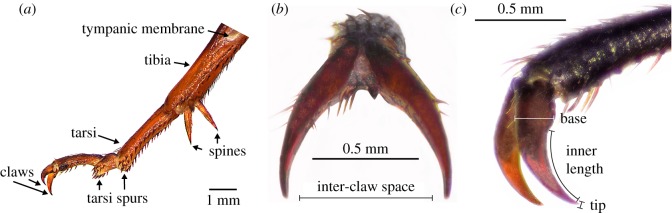
Cricket leg structure. (*a*) Image of a cricket leg. (*b*) Frontal view of the claws, indicating the measured inter-claw distance. (*c*) Lateral view of the frontal claws, indicating the measurements obtained (*n* = 10): claw base, claw tip and inner claw length.

The dimensions of female claws (*N* = 10; figure 3; electronic supplementary material, table D1) were measured with a Leica DFC495 digital camera coupled to a Leica M125 stereomicroscope, controlled by Leica Application Software (LAS) Ver. 3.8.0.

### 2.4. Trackball surfaces

**Figure 4.**
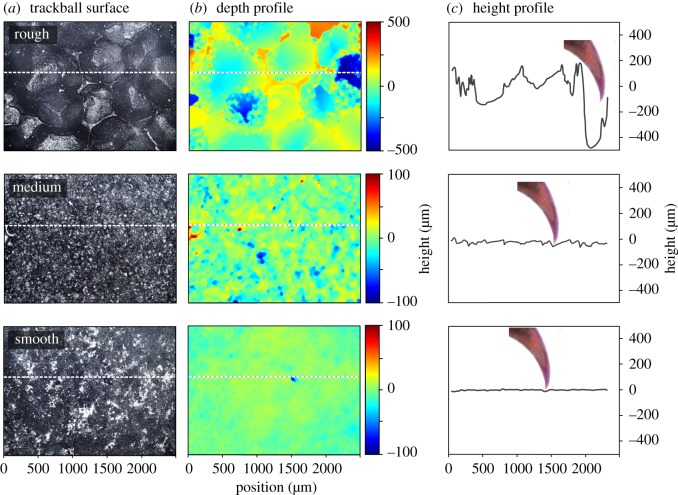
Profilometer measurements of the rough, medium and smooth trackballs. (*a*) Montage images of the surfaces. The white dotted line indicates the transect that was used for a depth profile of the surface. (*b*) Depth profile of the three trackball surfaces. The colour scale given at the right relates to the surface texture, with red corresponding to peaks and blue to troughs. Note a different colour scale is used for the medium and smooth trackball to allow a better colour coding of the texture. (*c*) The dark-grey line indicates the height profile of the surface for the given transect. A claw tip is presented at the same scale in order to allow comparison with the height profile.

Two types of Rohacell (Evonik, Rohacell, Germany) with different diameters of the methyl-methacrylate foam cells (Rohacell 31 HF: 150 µm, Rohacell 31 IG-F: 800 µm) were used to machine the trackballs, resulting in different surface pores (data as supplied by the manufacturer; figure 4; electronic supplementary material, table E1). Rohacell is manufactured to industrial and military standards by Evonik. All trackballs were sprayed with a matte black paint (Stove paint, flat black, Calfire Stove Bright) as a dark surface is necessary for the optical sensor to detect the rotation of the trackball. This reduced the pore size; the Rohacell 31 HF trackball with 150 µm cells was additionally sprayed with conductive black paint (Graphite, conductive coating, Kontakt Chemie, Iffezheim, Germany), which covered the pores of the 31HF material and generated a smooth surface. Given that the trackball floats in an airstream, the roughness of the surface remains stable; there is no wear and tear during the experiments.

All trackballs had a diameter of 5.6 cm, and a weight of 3.7 g, 3.3 g and 5.0 g for the smooth, medium and rough trackballs, respectively (electronic supplementary material, table E1). Control experiments were performed to determine whether the weight had an effect on the walking of the females (electronic supplementary material, table F1, F2 and Boxplot 1).

### 2.5. Profilometry measurements

Profilometry measurements were used to characterize the different trackball surfaces to obtain a surface height profile describing the width and depth of pores, i.e. the *R*_q_ roughness. The mean *R*_q_ roughness is a measure of the height variation in the sample, calculated as the RMS height averaged over the given area. An Olympus BX51 optical microscope with 5× objective was used to measure *z*-stack images of the trackballs. Montage images of the surfaces (figure 4) were constructed within Olympus LAS software by merging the image stacks. Height maps of the trackball surfaces were calculated from the image stacks using bespoke Matlab software, following the shape-from-focus technique [23]. The method selects, for a given pixel in the image, the image from a *z*-stack which has the best degree of focus, to give the height maps (figure 4*c*). The curvature on the ball was subtracted from the profile in the roughness analysis by first approximating the long-wavelength profile in the imaged area using a fitted fourth-order polynomial surface, and then subtracting this from the original data to retain only the shorter wavelengths appropriate to the scale of the contact with the insect. The *R*_q_ roughness for each of the imaged areas of size 2370 × 1780 µm was then evaluated on the roughness profile after subtracting this long-wavelength waviness, and the means of five such measurements per trackball were calculated.

### 2.6. Video recordings

Videos of cricket phonotactic walking on the three trackball surfaces were recorded with an Olympus TG4 camera (electronic supplementary material, Videos S1 and S2).

### 2.7. Statistical analysis

Data were analysed in R software (Ver. 3.2.2). We used Wilcoxon signed-rank test to quantify whether trackball texture had an impact on the phonotactic walking response, and to compare the contact force measurements. These Wilcoxon signed-rank tests were used given that the data were not normally distributed. We used a two-sided *t*-test to compare the roughness data for the three surfaces. A *t*-test was used because we were comparing whether one set of roughness data is bigger or smaller than another.

## 3. Results

During phonotactic tests, we had noticed that the behaviour of female *G. bimaculatus* varied on different trackballs. We subsequently measured systematically their response when walking on three trackballs, each having a different surface texture.

### 3.1. Analysis of phonotactic walking behaviour

When placed on a trackball and exposed to the male calling song, female crickets will walk towards the active speaker (figure 1). The phonotactic orientation to the active speaker is represented in the triangle-like lines giving the lateral deviation of the walking female (figure 1*b*). The rectangles represent the sound stimuli from the left and right. When the left speaker is active the cricket walks towards the left, and vice versa. On a smooth surface the crickets slide their feet on the trackball to obtain a mechanical contact point for the claws and the stepping pattern becomes very irregular; the phonotactic response was only 6.7 ± 1.2 cm min^−1^ and significantly lower than the other responses (***p < 0.001; figure 1*c*; electronic supplementary material, tables A1 and A2). On the medium and rough surfaces, the claws can be interlocked with the substrate allowing friction. When the leg touches the trackball at the end of the swing phase it interlocks with the trackball surface, and the subsequent stance phase allows pushing the body forward for locomotion. On the medium texture, crickets walked 36.6 ± 2.0 cm min^−1^, significantly faster than on the smooth texture. On the rough trackball, the crickets' walk was fast and constant, presenting the highest phonotactic response with 43.9 ± 9.4 cm min^−1^ (figure 1*c*; electronic supplementary material, tables A1 and A2). In conclusion, a smooth surface prevents crickets from walking properly, resulting in a weak phonotactic response, but on a medium or rough surface texture females showed a robust phonotactic response (electronic supplementary material, Video S1). Despite the rough trackball being the heaviest, the phonotactic response was larger than with the medium and smooth trackballs. Control experiments demonstrated that the weight difference between 3 g and 5 g trackballs does not significantly affect walking of the females (electronic supplementary material, table F1, F2 and Boxplot 1).

### 3.2. Claws and contact force measurements

We subsequently conducted contact force measurements between the crickets' feet and the trackball surfaces in order to understand the differences in walking behaviour. Females were tethered at the thorax to a strain gauge (figure 2*a*) and positioned on a trackball. While the insects were standing on the trackball the force to overcome the contact between legs and trackball surface was measured by moving the trackball away from the animal until the female could no longer hold on to the surface (see Material and methods).

On the smooth trackball, which lacked pores, the contact force (2.4 ± 0.1 mN) was significantly lower (***p < 0.001; figure 2*b*; electronic supplementary material, tables B1 and B2, Video S2) when compared with the forces generated on the medium and the rough trackball surfaces. On the rough and medium textures, with abundance of pores, the claws could be locked into the surface to grasp the trackball. The contact force generated on the medium surface was 12.2 ± 0.4 mN and on the rough surface was 23.9 ± 0.7 mN (figure 2*b*); these values were significantly different (***p < 0.001; figure 2*b*; electronic supplementary material, tables B1 and B2, Video S2).

Insect tarsi and claws have a major role in walking [24]. Cricket claws are positioned at the front of the tarsi and are arranged in a pair (figure 3). As claws interlock with the substrate, we measured their physical dimensions in order to compare the size of the claws with the surface texture of the trackballs. The pores of the rough and medium surfaces are similar in size to the claw tips of the crickets (figure 4*c*; electronic supplementary material, tables D1 and E1). The claws have a pointed hook-like structure, and the average diameter of the claw tip (figure 3*c*) of the front, middle and hind leg was 11.7 ± 0.4, 12.0 ± 0.3 and 11.9 ± 0.3 µm, respectively. The distance between the claws, i.e. the inter-claw space (figure 3*b*), of the front, middle and hind leg was 730.5 ± 13.6, 770.5 ± 15.2 and 804.6 ± 11.8 µm, respectively. Overall, the inter-claw distance was slightly larger on the hind claws. The average size of the claw inner length (figure 3*c*) of the front, middle and hind leg was 589.7 ± 9.5, 602.2 ± 21.6 and 760.0 ± 1.9 µm, respectively, the claw inner length being slightly larger on the hind claws. The average diameter of the base claw (figure 3*c*) of the front, middle and hind leg was 267.1 ± 6.0, 256.7 ± 2.7 and 278.8 ± 2.9 µm, respectively.

In order to understand the role of the claws when generating contact force, we removed the claws in 10 females, but otherwise the tarsi remained intact. The contact force in clawless crickets decreased significantly to 1.2 ± 0.1 N, even on the rough trackball (***p < 0.001; figure 2*b*; electronic supplementary material, tables B1 and B2), and was similar to the force of an intact cricket on the smooth trackball (*p* < 0.16; figure 2*b*; electronic supplementary material, tables B1 and B2). Intact crickets generated the strongest contact on the rough (23.9 ± 0.7 N) and medium (12.2 ± 0.4 N) trackball textures. The weakest contact was generated on the smooth trackball texture (2.4 ± 0.1 N) by intact crickets and on any surface by crickets with the claws removed.

### 3.3. Profilometry measurements

Profilometry measurements of the trackballs provide a characterization of the surface roughness and the depth of the pores (figure 4). The mean amplitudes of the *R*_q_ roughness for the smooth, medium and rough surfaces were 7.3, 16 and 180 µm, respectively, with *t*-tests showing significant differences between these values (*p* < 0.001; electronic supplementary material, tables E1 and E2). Thus, the rough surface had the largest and deepest pores as expected, whereas the smooth surface had a medium surface with hardly any pores (figure 4*b*). In order to compare the size of the claw tip diameter to the roughness of the three textures, a trackball surface height profile was generated and combined with a proportionate sized claw to demonstrate the interlocking capabilities (figure 4*c*). On the rough surface, the pore depth allows the claw a proper interlock with the substrate. On the medium surface, the pore depth allows a reduced contact force with the substrate. Finally, the smooth surface does not offer pores that allow a proper contact between the claw tip and the trackball surface and therefore does not allow generation of a proper contact force (figure 4*c*).

## 4. Discussion

Leg structures in insect species have evolved as an adaptation of locomotion on specific substrates [25]; claws, medium adhesive pads, hairy adhesive pads and multi-jointed legs are some adaptations in walking insects [2,4,26–28]. In female crickets, *G. bimaculatus*, we found that the surface structure is crucial for the phonotactic walking response.

### 4.1. Cricket leg adaptations

Substrate texture plays a key role in insect walking and in friction force generation [24]. Insects such as beetles [24] and crickets are adapted to walk on a rough substrate. Unlike other insects, which present medium or hairy attachment pads, adapted to climb vertically and to walk on slippery surfaces [27,29,30], the feet of ground-living insects are composed of movable claws, tarsal spurs and spines [6], which can be adapted in position to the substrate conditions.

The tarsi of crickets lack specialized adhesive pads, thus crickets are not efficient at walking on slippery surfaces. On a smooth surface, the stepping cycle becomes irregular as the crickets slide their feet on the trackball to obtain a mechanical contact point for the claws; the structure of the claw is important for this mechanical contact. On the rough surface, the claws can be interlocked with the substrate, because the claws can be inserted in the pores of the rough texture, which allows them to generate a high contact force. The subsequent stance phase pushes the body forward for locomotion. Claw interlock on a rough surface is also crucial for the jump of house crickets (*Acheta domesticus*); the longest jumps occurred with a higher friction, i.e. when the average particle diameter of the substrate was similar in size to the insect's claw diameter [31]. Finally, when the experiments were performed we only had the heavy rough trackball available. Owing to the difference in weight we later ran control experiments with a light-weight rough material to determine if weight had an effect on the walking behaviour. The control experiments (electronic supplementary material, tables F1, F2 and Boxplot 1) show that within the range tested weight did not have a significant effect on phonotactic steering. However, the surface texture always did affect significantly the walking response of the females. In addition, previous studies have used lighter and heavier trackballs to measure cricket walking responses: 12 cm diameter, 2.8 g [32]; 10 cm diameter, 12.8 g [33]; 8 cm diameter, weight not specified [34]. This demonstrates that crickets can walk on trackballs with different weights.

### 4.2. Contact force measurements

In the ground-living beetle, *Pachnoda marginata* [24], the relationship between the dimension of the claw tip and the substrate texture is crucial for grasping onto rough substrates [24]. Claws provide an anchor point for engagement of the foot so that the leg muscles can generate the force to move the insect across the ground. The ability of the claw tip to engage with the surface of the trackballs depends on the surface texture.

In our contact force experiment, the pore size of the rough and medium trackballs allowed proper interlock between the claw and the surface (figure 4*c*), whereas the smooth trackball presented less opportunity for the claws to engage or produce contact force and the very few pores available were smaller than the claw tip (electronic supplementary material, tables B1 and D1). This conclusion is based on scaling of the claw size and surface roughness, and is supported by the electronic supplementary material, Videos S1 and S2. In the case of *P. marginata* also, when the roughness of the surface was larger than the diameter of the claw tip, the beetle could grasp surface irregularities, and generate a high friction mechanical contact due to mechanical interlocking with the substrate texture. On the other hand, when the substrate presented pores of lower roughness amplitude compared with the claw tip diameter, the claws did not establish effective mechanical contact with the substrate, and as a consequence the beetle claws slipped over the surface. In our experiments, female crickets generated high contact forces on the rough surface. The large and relatively deep pores on rough trackballs (mean *R*_q_ roughness of 110 µm; electronic supplementary material, table E1) allowed the claw tip (mean 11.7 µm; electronic supplementary material, table D1) to attach through mechanical interlocking to the substrate, similar to the beetle example. In contrast, on the smooth surface the contact force was weak, because the claws could not be inserted into pores of the trackballs (mean *R*_q_ roughness of 5.3 µm; electronic supplementary material, table E1).

Removing the claws of an insect will change their walking and substrate attachment performance [27]. Crickets without claws do not walk as fast as intact crickets. Removal of the claws in the beetle *Gastrophysa viridula* led to a significant reduction in contact force when the insect was walking on rough substrates [27], even though the beetle presents hairy attachment pads. Thus, tarsal claws appear to be the essential functional structure for walking in ground-living insects.

## 5. Conclusion

In conclusion, the surface texture is a crucial parameter when testing walking responses of any animal that uses claws for walking or climbing, such as primates [35], birds [36] and the African clawed frog [37]. This is especially important for behavioural experiments of insects of any kind, including arena and trackball experiments [19,20,38–40], and needs to be considered in order to allow reliable comparisons between studies. For proper walking responses, crickets and probably other ground-living insects, which lack adhesive pads, need a rough surface to establish efficient anchor points for their claws. For phonotactic experiments, the surface texture of the trackball or of the arena needs consideration and should allow insertion of the tarsal claws for the generation of sufficient contact force. Finally, this study may provide information for engineers seeking to design agile legged insect-like robots [25,41,42] by understanding the conditions of insect locomotion on the ground.

## Supplementary Material

Tables A1, A2, B1 and B2;Video 1

## Data accessibility

Data are provided in the electronic supplementary material.
