# Supplementary material for: Substrate texture affects female cricket walking response to male calling song
Source: R Soc Open Sci. 2018 Mar 7;5(3):172334. doi: 10.1098/rsos.172334 (PMC5882743; doi:10.1098/rsos.172334)
Supplement: Tables A1, A2, B1 and B2;Video 1 [file rsos172334supp1.docx]

# Supplementary information

## A. Analysis of phonotactic walking behaviour

Table A1. Statistical summary showing the effects of the three trackball surfaces on female phonotaxis for all animals tested (n=25).

| Surfaces | Phonotaxis | |  |  |  |  |  |  |
| --- | --- | --- | --- | --- | --- | --- | --- | --- |
|  | **Mean**  **(cm/min)** | **Median**  **(cm/min)** | **SD** | **SEM** | **Min** | **1^st^ Qu.** | **3^rd^ Qu.** | **Max** |
| Smooth | 6.7 | 4.9 | 5.9 | 1.2 | 0.0 | 2.9 | 10.5 | 19.4 |
| Medium | 36.6 | 35.3 | 10.1 | 2.0 | 18.2 | 31.2 | 44.6 | 55.8 |
| Rough | 43.9 | 42.3 | 9.4 | 1.9 | 30.2 | 38.4 | 51.5 | 63.6 |

Table A2. Wilcoxon Signed Rank test comparing the effects of the three trackball surfaces on female phonotaxis. Bold data are highly significantly different.

| **Wilcoxon Signed Rank Test** | | | |
| --- | --- | --- | --- |
| **Surfaces** | **Smooth** | **Medium** | **Rough** |
| **Smooth** | **-** | **-** | - |
| **Medium** | **0.001***** | **-** | - |
| **Rough** | **0.001***** | **0.004**** | - |

**Video 1. Video of the same cricket walking on the three trackball surfaces. Note that in this particular video the female seemed to show a slight reduction of velocity on the medium trackball. However, the tested females walked faster on the medium trackball in comparison with the female of the video. On the smooth trackball the cricket uses all of its legs, but does not find proper contact points for any of the legs. The legs move independently in an uncoordinated manner on the smooth trackball.**

<https://drive.google.com/open?id=0Bzc1PYhVH-50WVRtQnQtMjN2bjg>

## B. Contact force measurements

Table B1. Statistical summary showing the contact forces generated by 10 female crickets on the three trackball surfaces, with claws and claws removed

| **Surface** | **Contact Forces** | |  |  |  |  |  |  |
| --- | --- | --- | --- | --- | --- | --- | --- | --- |
|  | **Mean**  **(mN)** | **Median**  **(mN)** | **SD** | **SEM** | **Min** | **1^st^ Qu.** | **3^rd^ Qu.** | **Max** |
| **Smooth (claws)** | 2.4 | 2.3 | 0.9 | 0.1 | 0.7 | 1.8 | 3.1 | 4.1 |
| **Medium (claws)** | 12.2 | 11.9 | 3.5 | 0.4 | 5.5 | 9.2 | 14.7 | 20.6 |
| **Rough (claws)** | 23.9 | 24.3 | 7.1 | 0.7 | 8.3 | 19.7 | 27.7 | 42.1 |
| **Rough (no claws)** | 1.2 | 1.2 | 0.4 | 0.0 | 0.6 | 0.8 | 1.5 | 1.8 |

Table B2. Wilcoxon Signed Rank test comparing the contact forces generated by 10 female crickets on the three trackball surfaces, with claws and claws removed (rough). Bold data are highly significantly different.

| **Wilcoxon Signed Rank Test** | | | | |
| --- | --- | --- | --- | --- |
| **Surfaces** | **Smooth (claws)** | **Medium (claws)** | **Rough (claws)** | **Rough (claws removed)** |
| **Smooth (claws)** | - | - | - | - |
| **Medium (claws)** | **0.001***** | - | - | - |
| **Rough (claws)** | **0.001***** | **0.001***** | - | - |
| **Rough (clawless)** | **0.001***** | **0.001***** | **0.001***** | - |

**Video 2. Video of the contact force measurements of the same female cricket standing on different trackballs. For this demonstration video the trackball was moved gradually over a time period of 20-30 s. During the actual force measurement the detachment was tested by manually moving the micromanipulator 2.3 cm in less than one second.**

<https://drive.google.com/open?id=0Bzc1PYhVH-50QTJpZi02TV9TRVU>

## C. Calibration measurements: Contact force


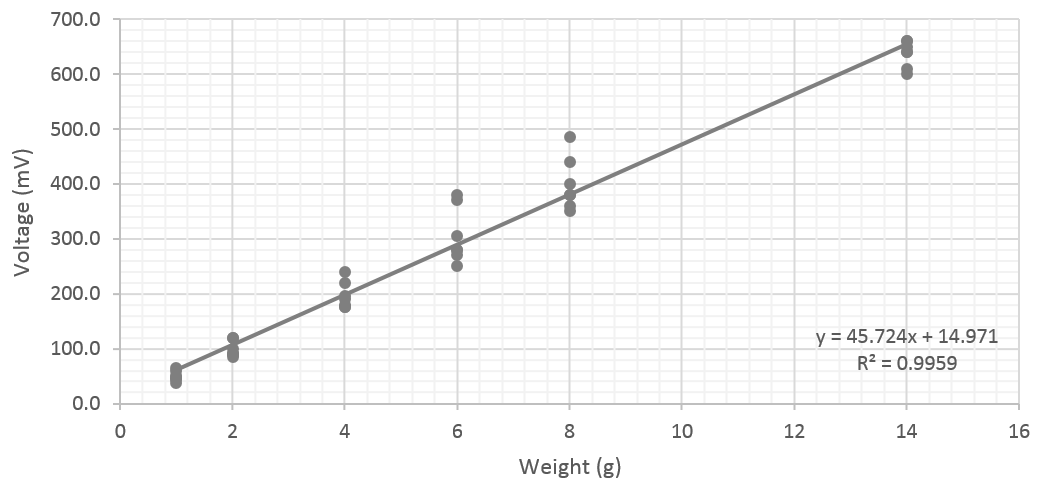


Figure 1. Linear regression of the strain gauge calibration, tested six times for each weight.

## D. Claws measurements

Table D1. Statistical summary showing the claw measurements of 10 female crickets.

| Claws | Mean  (mm) | Median  (mm) | SD | SEM | Min | 1^st^ Qu. | 3^rd^ Qu. | Max |
| --- | --- | --- | --- | --- | --- | --- | --- | --- |
| Front Claws |  |  |  |  |  |  |  |  |
| Inter-claw space | 730.5 | 725.6 | 43.0 | 13.6 | 657.3 | 704.9 | 754.6 | 794.7 |
| Tip of the claw | 11.7 | 11.6 | 1.2 | 0.4 | 10.0 | 10.9 | 12.6 | 13.7 |
| Base of the claw | 267.1 | 268.1 | 19.5 | 6.2 | 241.1 | 252.7 | 281.0 | 295.9 |
| Claw inner length | 589.7 | 592.0 | 30.1 | 9.5 | 538.6 | 571.0 | 607.0 | 634.6 |
| Middle Claws |  |  |  |  |  |  |  |  |
| Inter-claw space | 770.5 | 775.6 | 48.1 | 15.2 | 687.5 | 758.1 | 782.8 | 853.1 |
| Tip of the claw | 12.0 | 12.0 | 1.0 | 0.3 | 10.2 | 11.6 | 12.4 | 13.8 |
| Base of the claw | 256.7 | 256.9 | 8.4 | 2.7 | 244.9 | 251.8 | 259.4 | 275.6 |
| Claw inner length | 602.2 | 611.8 | 68.3 | 21.6 | 481.8 | 566.6 | 649.4 | 682.4 |
| Hind Claws |  |  |  |  |  |  |  |  |
| Inter-claw space | 804.6 | 812.6 | 37.3 | 11.8 | 745.2 | 773.9 | 827.4 | 869.6 |
| Tip of the claw | 11.9 | 12.0 | 1.0 | 0.3 | 10.3 | 11.4 | 12.4 | 13.6 |
| Base of the claw | 278.8 | 278.4 | 9.1 | 2.9 | 266.6 | 271.2 | 285.4 | 292.1 |
| Claw inner length | 760.0 | 759.5 | 6.1 | 1.9 | 752.2 | 756.4 | 761.9 | 771.4 |

## E. Trackball specifications and roughness

Table E1. Two trackball texture material and pore sizes provided by Rohacell. Mean and standard deviation of the R_q_ roughness for a series of five measurements on each of the three trackball surface profile measurements. These numbers can be quoted to two significant figures.

| **Surface** | **Material** | **Rohacell** | **Weight**  **(g)** | **Details** | **Roughness** | |
| --- | --- | --- | --- | --- | --- | --- |
|  |  | **Original pore size (µm)** |  |  | **Mean (µm)** | **SD**  **(µm)** |
| **Smooth** | Rohacell 31 HF | ~ 150 | 3.7 | Smooth surface covered with conductive paint decreasing the pore size to 40-80 µm | 7.3 | 1.3 |
| **Medium** | Rohacell 31 HF | ~ 150 | 3.3 |  | 16 | 1.5 |
| **Rough** | Rohacell 31 IG-F | ~ 800 | 5.0 |  | 180 | 11 |

Table E2. Two sided unpaired T-test to compare the surface texture of the three trackballs. The assumption that the two populations R_q_ heights are independent random samples from the same population with normal distributions with equal means and variances is rejected at a significance level of 0.01 in all three cases.

| **Two sided T-test** | |
| --- | --- |
| **Surfaces** | **P** |
| **Smooth vs Medium** | **<0.001***** |
| **Smooth vs Rough** | **<0.001***** |
| **Medium vs Rough** | **<0.001***** |

## F. Control experiment: comparison between trackballs of different weights

Table F1. Four trackballs were tested to compare the difference of weight effect on phonotactic behaviour.

| **Control experiment** | |
| --- | --- |
| **Surface** | **Weight (g)** |
| **Heavy trackballs** |  |
| **Heavy/Smooth** | 6.4 |
| **Heavy/Rough** | 5.3 |
| **Light trackballs** |  |
| **Light/Smooth** | 3.7 |
| **Light/Rough** | 3.3 |


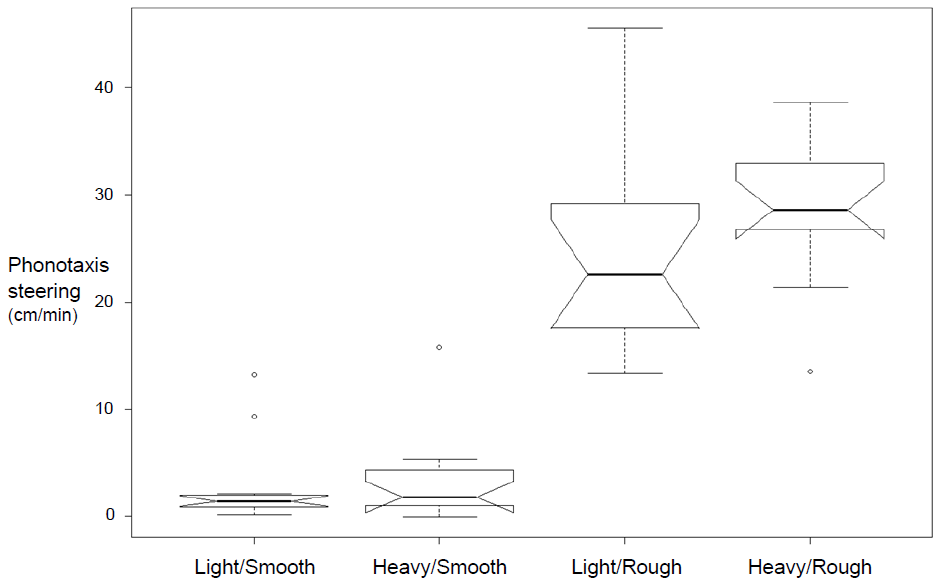


**Boxplot 1. Boxplot of the phonotactic behaviour of 10 female crickets on the four trackballs with different weights.**

Table F2. Wilcoxon Signed Rank test comparing the phonotactic response of 10 female crickets on the four trackballs with different weights. Bold data are highly significantly different.

| **Wilcoxon Signed Rank Test** | | | | |
| --- | --- | --- | --- | --- |
| **Surface** | **Light Smooth** | **Heavy Smooth** | **Light Rough** | **Heavy Rough** |
| **Light Smooth** | - | - | - | - |
| **Heavy Smooth** | 0.3 | - | - | - |
| **Light Rough** | **0.001***** | **0.001***** | - | - |
| **Heavy Rough** | **0.001***** | **0.001***** | 0.4 | - |
